# Whole-genome characterization and antibiotic resistance phenotype of *Escherichia marmotae* first isolated from *Berylmys bowersi*

**DOI:** 10.1128/spectrum.02946-24

**Published:** 2025-05-28

**Authors:** Ying Liu, Jian Zhou, Tao Gu, Weiwei Ai, Jingzhu Zhou, Qing Ma, Yong Hu, Shijun Li

**Affiliations:** 1 Guizhou Center for Disease Control and Prevention, Key Laboratory of Microbio and Infectious Disease Prevention and Control in Guizhou Province, Guiyang, China; 2 School of Public Health, the Key Laboratory of Environmental Pollution Monitoring and Disease Control, Ministry of Education, Guizhou Medical University, Guiyang, China; College of New Jersey, Ewing, New Jersey, USA

**Keywords:** *Escherichia marmotae*, *Berylmys bowersi*, phylogenetic analysis, antibiotic resistance, virulence factors, whole-genome sequencing, zoonotic potential, wildlife pathogens, microbiology

## Abstract

*Escherichia marmotae* was first described in 2015 as a bacterium isolated from Himalayan marmots, with recent evidence suggesting its potential to cause human diseases. This study presents the first report of *E. marmotae* isolation from *Berylmys bowersi* in Guizhou province. We conducted genetic, biochemical, and antibiotic resistance analyses on four isolates, designated as S2-2, S2-4, S2-5, and S2-6. Phylogenetic analysis based on 16S rRNA sequences revealed over 99.5% homology with *E. marmotae*, while pulsed-field gel electrophoresis and whole-genome sequencing confirmed genetic similarity. Gene annotation highlighted the presence of 137 virulence factors and five antibiotic resistance mechanisms, including resistance to fluoroquinolones and tetracyclines. Resistance phenotypes of 35 antibiotics showed resistance to penicillin, erythromycin, rifampin, and co-trimoxazole. The strains exhibited significant biochemical diversity, with positive results for several fermentation pathways and negative motility assays. This study underscores the emerging zoonotic potential of *E. marmotae* and its associated health risks in wildlife and humans.

**IMPORTANCE:**

The isolation of *Escherichia marmotae* from *Berylmys bowersi* represents a novel discovery, expanding the known host range of this bacterium. Our comprehensive analysis of its genetic, biochemical, and antibiotic resistance profiles provides critical insights into its potential as a zoonotic pathogen. The findings highlight the need for ongoing surveillance of *E. marmotae*, especially in wildlife populations, to assess its pathogenicity and potential threats to both animal and human health. Given its antibiotic resistance and virulence factor repertoire, *E. marmotae* may pose a significant public health concern in the future, warranting further investigation into its ecological and clinical relevance.

## INTRODUCTION

*Escherichia marmotae* was isolated from the intestinal contents of Himalayan marmots (*Marmota himalayana*) on the Qinghai-Tibet Plateau and was first described by Liu et al. in 2015 (1). *E. marmotae* belongs to the genus *Escherichia* within the family Enterobacteriaceae, class Gammaproteobacteria, and order Enterobacterales. The genus *Escherichia* is one of the most extensively studied groups in medical microbiology. To date, six species have been identified within this genus, including *Escherichia coli*, *Escherichia hermannii* (2), *Escherichia vulneris* (3), *Escherichia fergusonii* (4), and *Escherichia albertii* (5), all of which were established prior to the discovery of *E. marmotae*. Notably, *E. marmotae* is a newly identified member of the genus. A strain of *E. marmotae* was also isolated from the uterine purulent discharge of a 10-year-old female dog in Tianjin (6). In 2022, Sivertsen and colleagues isolated *E. marmotae* from four human patients, demonstrating that it may be a potential causative agent of human diseases and highlighting the possibility of misidentification of *E. marmotae* as *E. coli* (7). At the same time, Sinha and colleagues also clearly indicated the similarity between *E. marmotae* and *E. coli* in clinical practice (8). Since the discovery of *E. marmotae*, researchers have identified them in different hosts, investigating their antibiotic resistance and pathogenicity, with most studies relying on whole-genome sequencing (WGS) to predict both their resistance and potential pathogenicity (9–11).

To date, no studies have reported the isolation of *E. marmotae* from *Berylmys bowersi*, nor have there been investigations into the antibiotic resistance phenotypes, molecular typing, or biochemical characteristics of *E. marmotae* isolated from *Berylmys bowersi*. Therefore, this study represents the first report of *E. marmotae* isolation from *Berylmys bowersi* and includes an in-depth analysis of genetic identification, molecular typing, and drug resistance profiles of the strain.

## MATERIALS AND METHODS

### Strain sources

Rodent surveillance was conducted in Liping County, Guizhou Province, using the night trap method specified in GB/T 23798–2009, Monitoring Methods for Rodent Density of Vector-Borne Diseases (12). Traps (corn and peanuts as bait) were set overnight and retrieved the following morning. Captured mice were euthanized by cervical dislocation under ether anesthesia (ether purity >99%, exposed to a closed container for 10 minutes to confirm no pain reflexes) to ensure a rapid and painless process, in compliance with all relevant wildlife protection laws. During the survey, *Berylmys bowersi* was captured, and its species, size, and gender were identified according to the *Identification Handbook of Important Medical Animals in China* (13). Immediately following identification, small intestinal and rectal specimens (4 mm in size) were surgically excised under aseptic conditions and preserved in brain-heart infusion + 20% glycerol preservation solution. Primary isolation was performed on standard blood agar plates under aerobic conditions at dual incubation temperatures (28°C and 37°C) for 48 hours. Colonies exhibiting morphological consistency in size, hemolytic patterns, and pigmentation were selectively subcultured three times (1).

### Gene identification and molecular typing

The bacterial strains were isolated and purified via the streak plate method. Genomic DNA was subsequently extracted using a commercial nucleic acid extraction kit (Baiyi Biotechnology, Hangzhou, China) following the manufacturer’s standardized protocols (http://www.baiyi-tech.cn/). The 16S rRNA gene was amplified by PCR with primers 27F (5′-AGTTTGATCMTGGCTCAG-3′) and 1492R (5′-AGTTTGATCMTGGCTCAG-3′), followed by sequencing (14, 15). The total reaction volume was 50 µL, which included 25 µL of Premix Taq, 2 µL of each primer, 2 µL of template DNA, and 19 µL of water. The PCR amplification conditions were as follows: initial denaturation at 94°C for 10 minutes; 40 cycles of denaturation at 94°C for 45 seconds, annealing at 52°C for 60 seconds, and extension at 72°C for 60 seconds, followed by a final extension at 72°C for 10 minutes. The sequencing was then completed by Tianyi Huiyuan Biotechnology Co., Ltd (http://101.43.170.175/tyhy/). The sequences obtained were compared to the BLAST database at National Center for Biotechnology Information (NCBI), and closely related sequences from GenBank were downloaded for evolutionary analysis of the bacterial genus.

Pulsed-field gel electrophoresis (PFGE) was used for molecular typing, employing the XbaI restriction enzyme according to the *National Pathogen Identification Network Technical Manual* (2020 edition) and the protocol described in Dr. Liu Sha’s thesis (11). Freshly isolated strains were embedded in 1% Seakem Gold agar blocks. The bacteria were lysed and digested with XbaI for >2 hours, and the resultant DNA fragments were subjected to electrophoresis using *Escherichia coli* American Type Culture Collection (ATCC) 25922 as a quality control strain. The electrophoresis conditions were as follows: pulse time ranging from 2.2 to 54.2 seconds, with an electrophoresis run time of 19 hours. Gel images were captured using the Gel Doc 2000 system and analyzed with Bionumerics 8.0 software.

### WGS

A hybrid assembly pipeline integrating short-read (MGISEQ-2000RS) and long-read (Nanopore) sequencing data was implemented to generate a high-quality closed genome. The workflow comprised three phases: (i) dual-platform sequencing, (ii) raw data preprocessing, and (iii) iterative hybrid assembly optimization.

#### Sequencing platform configuration

##### Short-read sequencing

Libraries were prepared using the MGISEQ-2000RS FluoXpert platform (BGI, Shenzhen, China) with DNA nanoball (DNB) amplification technology (16). Genomic DNA was enzymatically fragmented (200–500 bp) using the MGIEasy DNA Library Prep Kit (BGI cat. no. 1000006983), ligated with dual-indexed adapters (BGI Universal Adapter Set v.4), and amplified through 15-cycle PCR. DNB arrays were sequenced in 2 × 150 bp paired-end mode following the manufacturer’s protocols.

##### Long-read sequencing

High-molecular-weight DNA (>20 kbp) was processed using the Ligation Sequencing Kit SQK-LSK109 (Oxford Nanopore Technologies, Oxford, UK). Fragments were end-repaired, adapter-ligated (Native Barcodes EXP-NBD104/114), and loaded onto R9.4.1 flow cells. Real-time sequencing was performed on the GridION X5 platform with MinKNOW v.21.05.1 basecalling (17).

### Data preprocessing

Raw MGISEQ reads underwent quality trimming using Trimmomatic v.0.39 (parameters: SLIDINGWINDOW:4:20 and MINLEN:50) (18). Nanopore reads were filtered (*Q*-score ≥7) and size-selected (>5 kbp) using Guppy v.5.0.7 (19). Cross-platform quality metrics were verified with FastQC v.0.11.9 (20) and NanoPlot v.1.32.1 (21).

### Hybrid assembly pipeline

The Micro IBS Analyzer platform (MicroFuture Tech, Beijing, China) orchestrated the following workflow: *de novo* assemblies: SPAdes v.3.15.4 (k-mer range: 21–127) (22) and Canu v.2.1.1 (corrected error rate = 0.045) (23) generated initial contigs from short-read and long-read data, respectively; iterative polishing: four-stage refinement using (i) Racon v.1.4.3 (Nanopore-aware consensus calling) (24), (ii) Medaka v.1.4.3 (ONT model r941_min_high_g360) (25), (iii) Pilon v.1.23 (Illumina-based single-nucleotide polymorphism/indel correction) (26), and (iv) Circlator v.1.5.5 (circularization verification) (27). Assembly completeness was validated through BUSCO v.5.2.2 (enterobacterales_odb10 data set) (28), achieving 98.6% single-copy ortholog recovery.

### Genetic analysis

Genome annotation was performed using the Beijing MicroFuture Pathogen Bioinformatics Analysis System platform, integrating queries against the following databases: Comprehensive Antibiotic Resistance Database (CARD) (29), Kyoto Encyclopedia of Genes and Genomes (KEGG) (30), Clusters of Orthologous Groups (COG) (31), NCBI Non-Redundant Protein Database (NR) (32), Pathogen-Host Interaction (PHI) database (33), and Virulence Factor Database (VFDB) (34). Circular genome maps were generated using the CGView Server (35).

Genomic homology and phylogenetic relationships were analyzed through average nucleotide identity (ANI) calculations against reference genomes in the EzBioCloud database (36), digital DNA-DNA hybridization (dDDH) via the Genome-to-Genome Distance Calculator v.3.0 (37), and 16S rRNA-based phylogeny constructed with the neighbor-joining method in MEGA v.11 (1,000 bootstrap replicates) (38).

### Biochemical characteristics and motility tests

Isolated strains and reference strains were inoculated onto Luria-Bertani agar plates using the streaking method and incubated at 37°C for 24 hours. Freshly cultured colonies were identified using an API 20E biochemical reagent strip (Merck) (39). In addition, strains were cultured on *Salmonella* chromogenic medium, *Salmonella*-*Shigella* agar selective medium, and MacConkey agar and incubated at 37°C for 24 hours (40, 41). Colony characteristics were observed, and Gram staining was performed on isolated colonies, followed by examination under a light microscope. Motility was assessed by inoculating strains into semisolid agar and performing a stab-inoculation method (42). After 24 hours of incubation at 37°C, motility was determined by the diffusion of the inoculation line: non-diffusive growth indicated negative motility, while a blurred, diffused line indicated positive motility.

### Antibiotic susceptibility testing

Following the guidelines established by the Clinical and Laboratory Standards Institute, the antimicrobial susceptibility of 35 antibiotics was evaluated using the Kirby-Bauer disk diffusion method and custom antimicrobial susceptibility testing plates (43, 44). These antibiotics were categorized primarily into the following classes: β-lactams, macrolides, fluoroquinolones, tetracyclines, sulfonamides, combination preparations, aminoglycosides, chloramphenicol, polycolistin, and other miscellaneous antibiotics using *E. coli* ATCC 25922 as a quality control strain.

## RESULTS

### Phylogenetic relationships of 16S rRNA gene sequences

**Fig 1:**
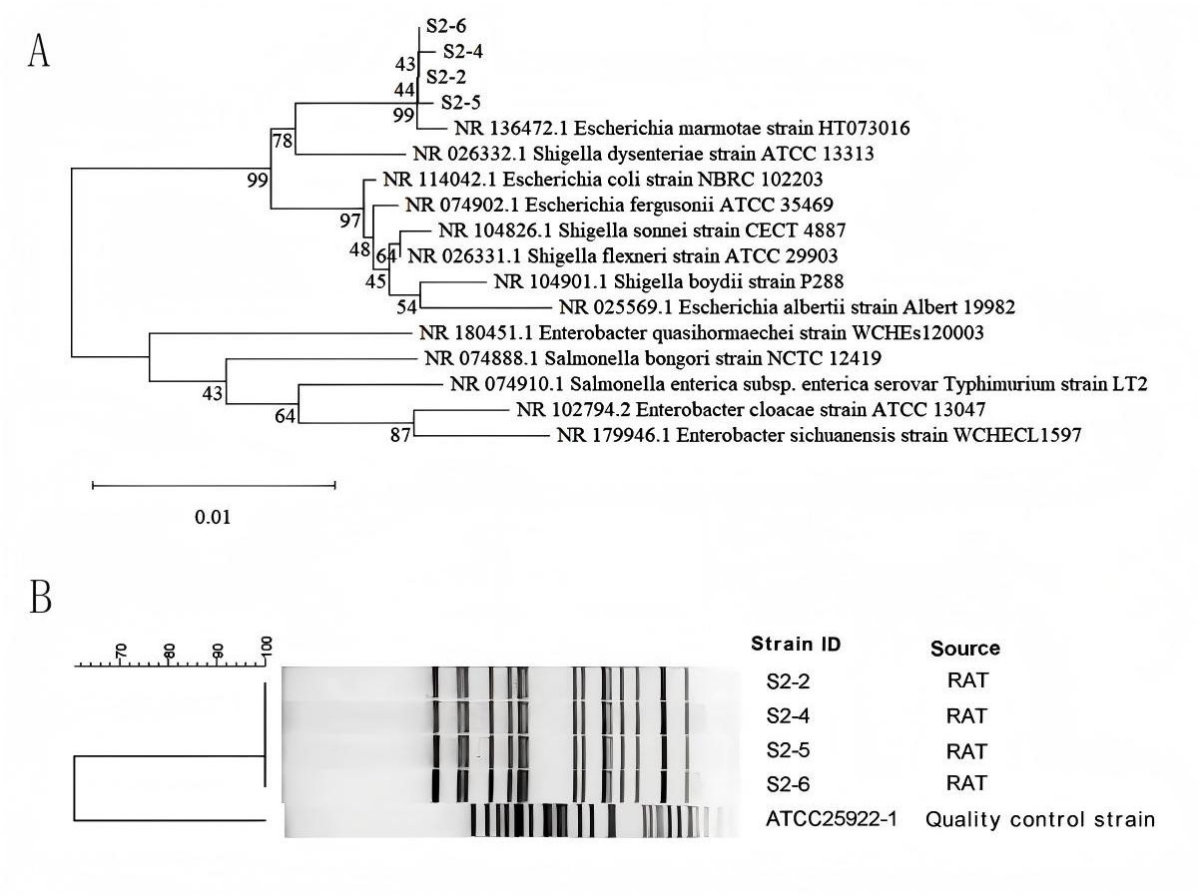
(**A**) Phylogenetic tree based on 16S rRNA gene sequences of strains S2-2, S2-4, S2-5, and S2-6 and 13 closely related strains. (**B**) PFGE analysis of strains S2-2, S2-4, S2-5, and S2-6.

Four morphologically similar bacterial strains were isolated from different segments of the intestines of *Berylmys bowersi* and designated as S2-2, S2-4, S2-5, and S2-6. The 16S rRNA gene sequences of these strains were compared against the NCBI database, and the top 13 closest matches were selected for further analysis. Phylogenetic analysis based on the constructed tree revealed that the sequences from the four isolated strains exhibited over 99.5% homology with *E. marmotae* strain NR_136472.1, indicating a close phylogenetic relationship. The strains also showed similarities with *Shigella dysenteriae* ATCC 13313, while clustering more distantly from other species within the *Escherichia*, *Shigella*, *Enterobacter*, and *Salmonella* genera (Fig. 1A).

### PFGE analysis

At the molecular level, PFGE analysis demonstrated that these four *E. marmotae* strains were genetically identical, with 100% similarity. In contrast, their genetic relationship with the *Escherichia coli* ATCC 25922 reference strain was more distant (Fig. 1B).

### WGS characteristics

**Fig 2:**
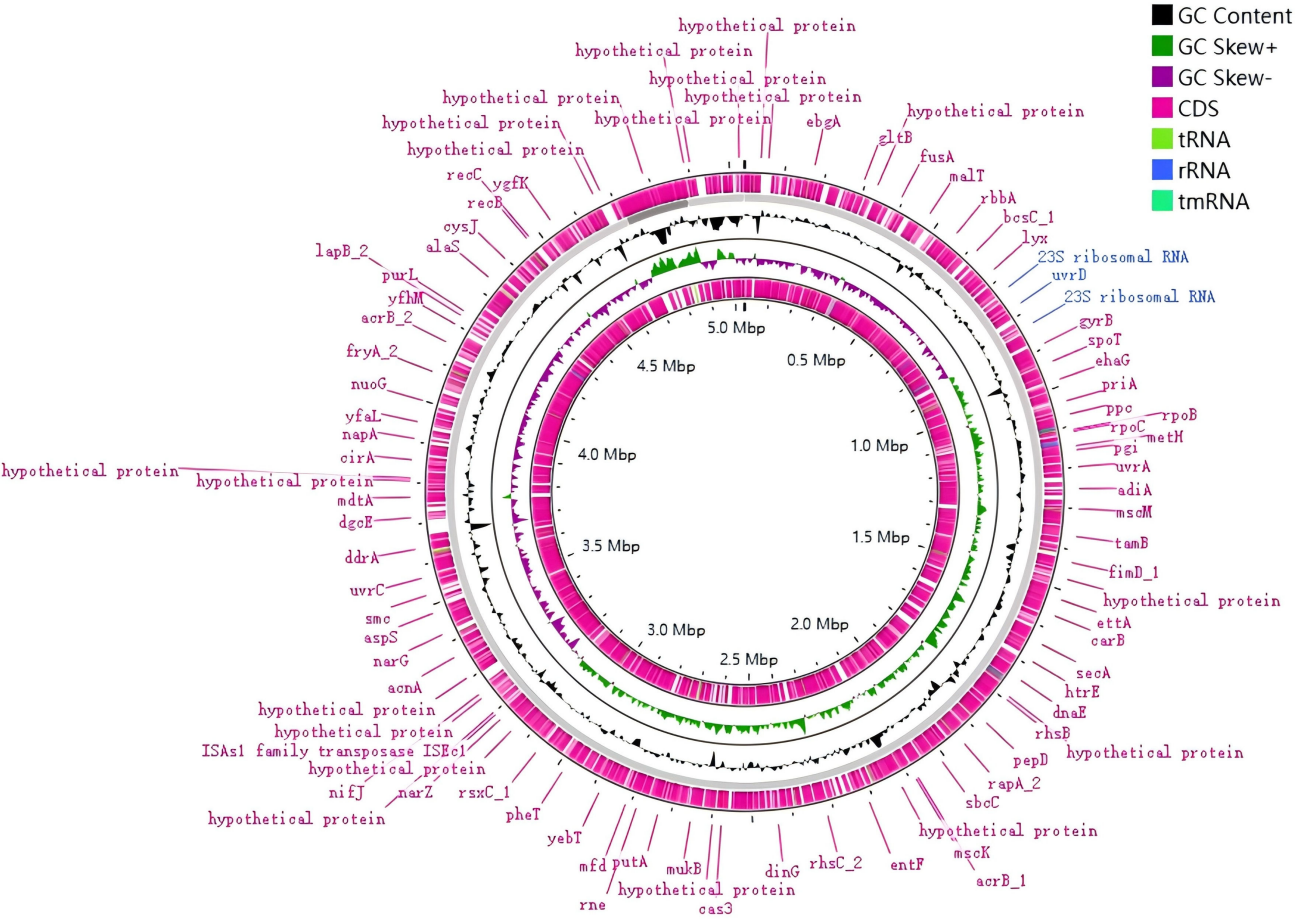
Complete genome map of strain S2-2. The map features a circular representation of the coding regions, color-coded by functional categories, including non-coding RNAs (tmRNA, tRNA, and rRNA), GC content and coding sequence (CDS), highlighting functional categories such as coding sequences, tRNAs, and rRNAs. Only a few gene names and their locations are shown.

WGS was performed on strains S2-2, S2-4, S2-5, and S2-6, with high consistency observed across the strains. Strain S2-2 was selected as the representative strain for further analysis. The combined second and third-generation WGS data for strain S2-2 yielded a genome of 5,037,959 bp with 99.91% integrity, 100% assembly quality, and 0.79% contamination. This genome contained 92 tRNA, seven copies of 23S rRNA, seven copies of 16S rRNA, and five copies of 5S rRNA. The guanine-cytosine (GC) content was 50.17%, and the N50 and N75 values were both 4,711,508 bp, indicating high-quality sequencing (Fig. 2). According to the evaluation criteria of high-throughput genetic data by Bowers et al. and Duan et al., this sequence was considered complete and of high quality (45, 46).

### Gene function analysis

Gene function annotation was performed using Prokka, resulting in the identification of 132, 4,063, 4,230, 4,680, 359, and 137 genes in the CARD, KEGG, COG, NR, PHI, and VFDB databases, respectively.

**Fig 3:**
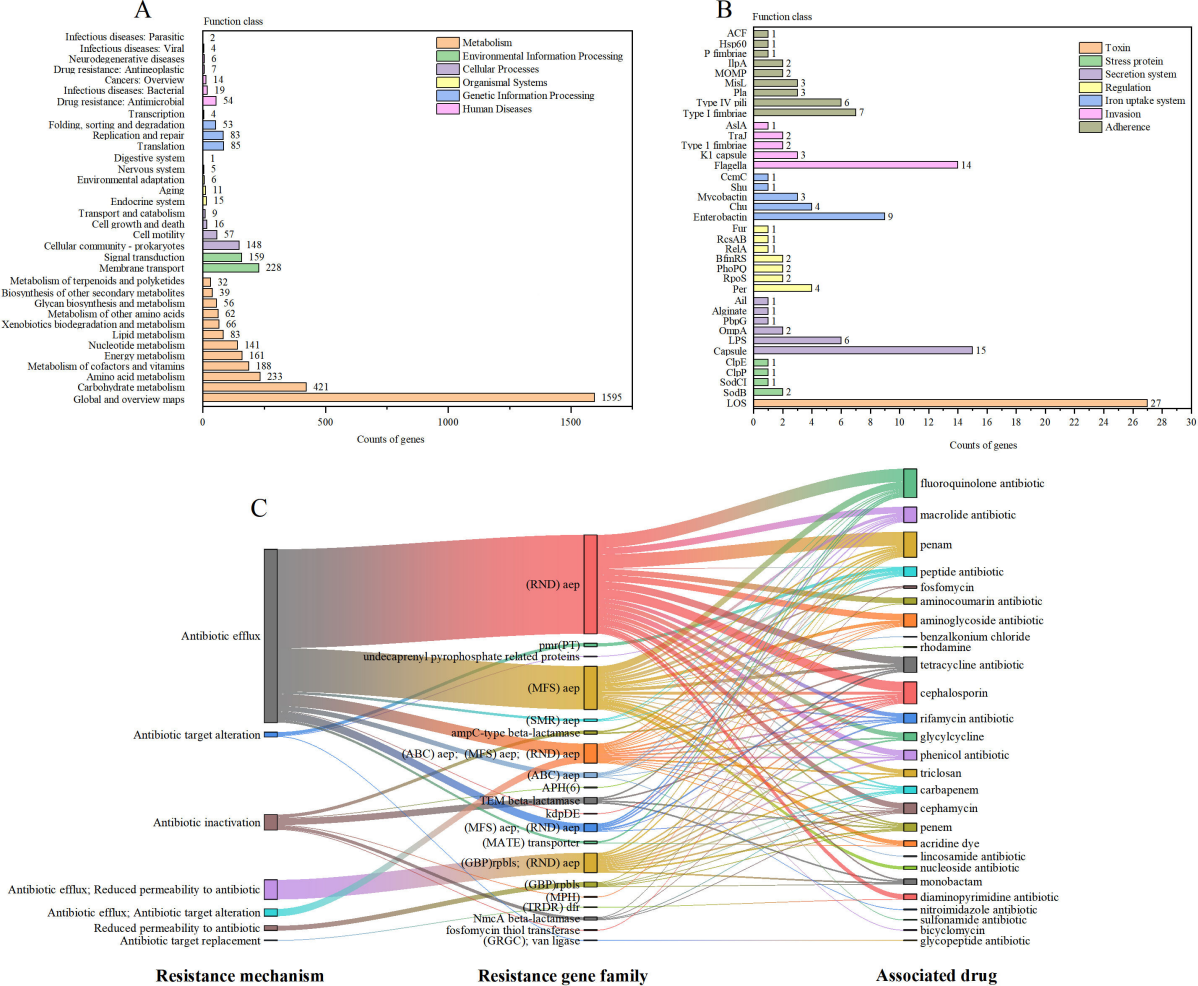
(**A**) KEGG pathway analysis of strain S2-2. (**B**) VFDB analysis of strain S2-2. (**C**) CARD analysis of strain S2-2. Abbreviations: ABC, ATP-binding cassette; AEP, antibiotic efflux pump; (GBP)rpbls, general bacterial porin with reduced permeability to beta-lactams; GRGC, glycopeptide resistance gene cluster; MATE, multidrug and toxic compound extrusion; MFS, major facilitator superfamily; MPH, macrolide phosphotransferase; PT, phosphoethanolamine transferase; RND, resistance-nodulation-cell division; SMR, small multidrug resistance; TRDR, trimethoprim-resistant dihydrofolate reductase.

KEGG pathway analysis revealed that 4,063 orthologous genes mapped to 34 metabolic pathways. The predominant categories were metabolism (35.3%), human diseases (20.6%), organismal systems (14.7%), cellular processes (11.8%), genetic information processing (11.8%), and environmental information processing (5.9%) (Fig. 3A). Notably, the human disease category showed a high concentration of genes associated with drug resistance and infectious diseases.

Virulence factor analysis using the VFDB identified 137 putative virulence genes in strain S2-2, with 36 genes having >80% identity (Table S1). These virulence factors were classified into seven categories: toxins (19.7%), adherence (19.0%), secretion system (19.0%), invasion (16.1%), iron uptake system (13.1%), regulation (9.5%), and stress proteins (3.6%) (Fig. 3B). Notably, genes associated with lipooligosaccharide, flagella, enterobactin, type I fimbriae, capsule, and lipopolysaccharide were the most abundant, which are critical for toxin production, invasion, iron uptake, adherence, and secretion systems.

In the CARD database, strain S2-2 exhibited five antibiotic resistance mechanisms: antibiotic efflux, reduced permeability, antibiotic inactivation, antibiotic target alteration, and antibiotic target replacement, covering 80 antibiotic resistance genes (132 gene segments); the identity value of these resistance genes exceeded 40%, among which 58 resistance genes had an identity value greater than 80% (Table S2). The strain was resistant to 27 antibiotics, with the highest resistance observed against fluoroquinolones, penam, cephalosporins, macrolides, and tetracyclines (Fig. 3C).

**Fig 4:**
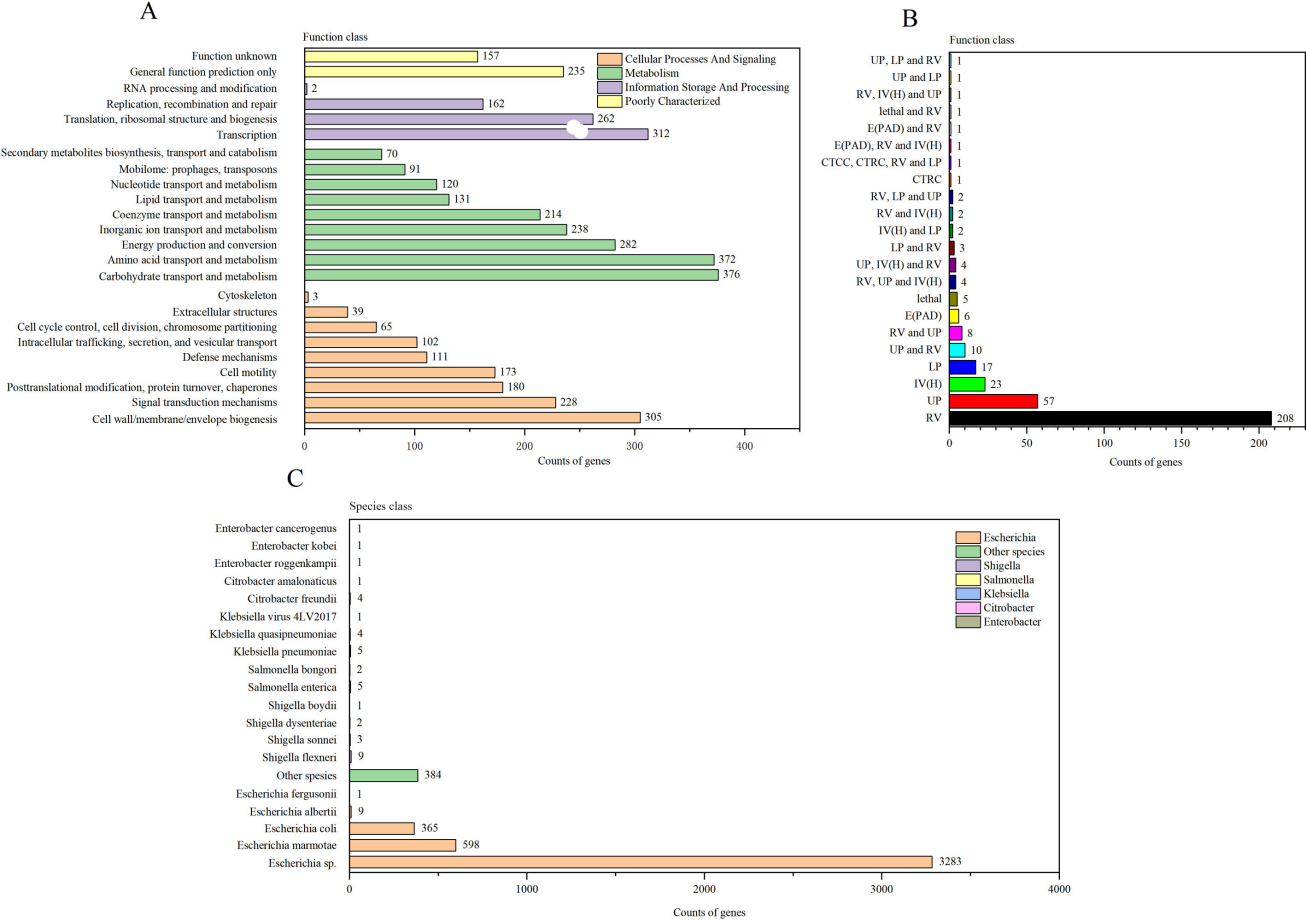
(**A**) COG analysis of strain S2-2. (**B**) Types distribution of PHI genes of strain S2-2. Abbreviations: CTCC, chemistry target: sensitivity to chemical; CTRC, chemistry target: resistance to chemical; E(PAD), effector (plant avirulence determinant); IV(H), increased virulence (hypervirulence); LP, loss of pathogenicity; RV, reduced virulence; UP, unaffected pathogenicity. (**C**) NR analysis of strain S2-2.

COG analysis annotated 4,230 genes, categorized into 24 functional groups. Major functional categories included carbohydrate transport and metabolism, amino acid transport and metabolism, transcription, translation, ribosomal structure and biogenesis, cell wall/membrane/envelope biogenesis, energy production and conversion, and inorganic ion transport and metabolism (Fig. 4A).

In the PHI database, 359 pathogen-host interaction-related genes were annotated. These were categorized into 22 groups, with reduced virulence (208 genes) being the most prominent, followed by unaffected pathogenicity (57 genes), increased virulence (23 genes), loss of pathogenicity (17 genes), and lethal (five genes) (Fig. 4B).

Analysis of the NR database revealed 4,680 annotated genes, with *Escherichia* spp. being the most frequently annotated, followed by *E. marmotae*. The number of *E. coli* annotations was second only to *E. marmotae* (Fig. 4C).

### WGS comparison: ANI and dDDH analysis

**Fig 5:**
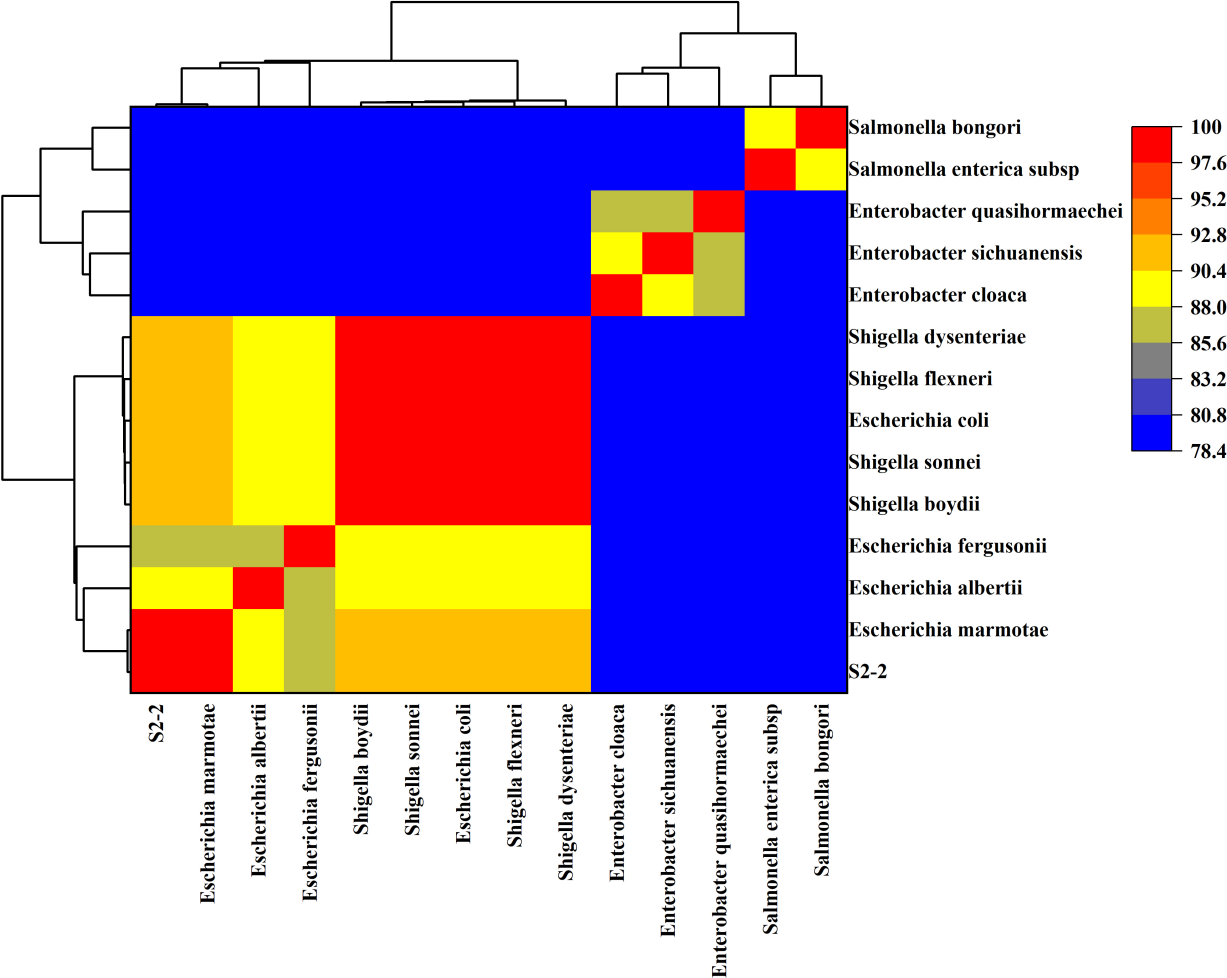
Heat map of ANI between strain S2-2 and 13 phylogenetically closely related species.

Strain S2-2 was selected for comparative analysis with 13 phylogenetically close species using ANI and dDDH. dDDH analysis revealed the highest similarity with *E. marmotae* (GCA_029962465.1) at 95.6% (dDDH ≥70%), with a probability of 97.35%. Other closely related species, including *Shigella sonnei*, *Shigella dysenteriae*, *E. coli*, and *Shigella boydii*, showed dDDH values below 70% (Table 1). ANI analysis further supported these findings, with an ANI value of 99.44% between strain S2-2 and *E. marmotae*, which is above the 95% threshold for high genomic similarity. Conversely, the ANI values with *E. coli*, *Shigella dysenteriae*, and other *Shigella* sp. were around 91%, further supporting the close relationship between strain S2-2 and *E. marmotae* (Fig. 5).

**TABLE 1:** dDDH values between strain S2-2 and 13 closely related species^*a*^

Reference genome	Formula 1		Formula 2		Formula 3	
	dDDH	Prob. dDDH ≥70%	dDDH	Prob. dDDH ≥70%	dDDH	Prob. dDDH ≥70%
*GCA_029962465.1_Escherichia marmotae*	84.4	94.61	95.6	97.35	89.1	99.29
*GCA_002950395.1_Shigella sonnei*	54.8	24.27	43.8	6.46	52.7	5.62
*GCA_022354085.1_Shigella dysenteriae*	62.6	51.28	43.8	6.51	59.2	21.92
*GCA_000005845.2_Escherichia coli*	63.8	55.73	43.7	6.31	60.2	25.94
*GCF_002290485.1_Shigella boydii*	61	45.55	43.6	6.22	57.8	16.99
*GCF_000006925.2_Shigella flexneri*	55	25	43.3	5.85	52.8	5.7
*GCA_028622335.1_Escherichia albertii*	59.3	39.57	38.4	1.78	54.6	8.69
*GCA_020097475.1_Escherichia fergusonii*	46.9	7.26	34.4	0.55	43.5	0.44
*GCF_000614485.1_Salmonella enterica* subsp. enterica serovar Typhimurium	29.4	0.07	23.5	0	27	0
*GCF_000252995.1_Salmonella bongori*	29.3	0.07	23.4	0	26.8	0
*GCF_905331265.2_Enterobacter cloacae*	24.5	0.01	22.9	0	23.1	0
*GCF_009036245.1_Enterobacter sichuanensis*	25.5	0.01	22.5	0	23.8	0
*GCA_004331385.1_Enterobacter quasihormaechei*	24.5	0.01	22.3	0	23	0

^*a*^ Prob., probability.

### Antibiotic resistance phenotype

Antibiotic resistance testing was performed against 35 antibiotics. All four *E. marmotae* strains exhibited resistance to penicillin, erythromycin, rifampicin, and co-trimoxazole, with intermediate resistance to two other antibiotics. The strains were sensitive to the remaining 29 antibiotics (Table 2).

**TABLE 2:** Antibiotic susceptibility testing results for strain S2-2^*a*^

Drug susceptibility test	Drug name	Drug abbreviation	Specification (μg)	Results	Drug susceptibility test	Drug name	Drug abbreviation	Specification (μg)	Results
KB	Rifampicin	RD	5	R	DSP	Ceftazidime/clavulanic acid	T/C	0.25/4.0–16/4	S
KB	Penicillin	P	10	R	DSP	Ceftazidime/avibactam	CZA	0.5/4.0–16/4	S
KB	Erythromycin	E	15	R	DSP	Ceftazidime	TAZ (CAZ)	0.5–32.0	S
KB	Co-trimoxazole	SXT	25	R	DSP	Cefotaxime/clavulanic acid	F/C	0.06/4.0–4/4	S
KB	Minocycline	MN	30	S	DSP	Ceftiofur	XNL	0.5–16.0	S
KB	Meropenem	MRP	10	S	DSP	Cefuroxime	FUR	1–64	S
KB	Levofloxacin	LEV	5	S	DSP	Cefepime	FEP	1–32	S
KB	Ciprofloxacin	CIP	5	S	DSP	Tigecycline	TGC	0.25–8.0	S
KB	Chloramphenicol	C	30	S	DSP	Tetracycline	TET	1–32	S
KB	Ceftriaxone	CRD	30	S	DSP	Gentamicin	GEN	1–32	S
KB	Cefotaxime	CTX	30	S	DSP	Nalidixic acid	NAL	2–64	S
KB	Azithromycin	AZM	15	S	DSP	Florfenicol	FFN	1–32	S
KB	Ampicillin	AMP	10	S	DSP	Ertapenem	ETP	0.25–8.0	S
DSP	Streptomycin	STR	4–32	I	DSP	Colistin	COL	0.25–8.0	S
DSP	Polymixin	POL	0.25–8.0	I	DSP	Ampicillin/sulbactam	A/S2 (AMS)	1.0/0.5–64/32	S
DSP	Imipenem	IMI (IPM)	0.25–8.0	S	DSP	Amoxicillin/clavulanic acid	AUG2	1.0/0.5–64/32	S
DSP	Cefazolin	FAZ	1–32	S	DSP	Amikacin	AMI	4–64	S
DSP	Cefoxitin	FOX (CFX)	1–64	S					

^*a*^ DSP, drug-sensitive plate; I, intermediary; KB, means Kirby-Bauer; R, resistant; S, sensitive.

### Biochemical characterization and motility testing

The four bacterial strains were consistently gram-negative short rods, occurring singly or in pairs with blunt, rounded ends. Morphological examination on Sha’s chromogenic medium, SS selective medium, and MacConkey agar revealed typical colony appearances, with white colonies on Sha’s medium, medium-pink colonies on SS medium, and pink colonies with some diffusion on MacConkey agar.

Motility assays were negative for all isolates, while the control strains *E. coli* ATCC 25922 and *Salmonella* Typhi were positive for motility.

Biochemical tests revealed that the isolates were positive for lysine decarboxylase, ortho-nitrophenyl-β-galactosidase (ONPG), indole (IND) production, and arginine dihydrolase, and negative for ornithine decarboxylase, tryptophan deaminase, urease, and gelatinase. The isolates fermented arabinose, melibiose, rhamnose, and glucose but did not ferment sucrose or amygdalin. They hydrolyzed mannitol and sorbitol but did not hydrolyze inositol, and they utilized acetate (Voges-Proskauer [VP]) while not utilizing sodium citrate. The triple sugar iron test indicated no H2S production, with acid and gas formation from lactose and glucose (Table S3).

## DISCUSSION

The isolation of *E. marmotae* from *Berylmis bowersi* adds to the growing evidence of the broad host adaptability of this species. *E. marmotae* was first identified from the intestinal contents of Himalayan marmots (*Marmota himalayana*), a member of the same order (Rodentia) as *Berylmis bowersi*. However, its subsequent detection in phylogenetically distant hosts, including dogs and humans (6, 7), highlights its remarkable ability to colonize diverse mammalian species. This study provides the first report of *E. marmotae* in *Berylmys bowersi*, a species endemic in Guizhou province. The results presented herein underline the importance of this bacterium as a potential zoonotic pathogen and the necessity of continued surveillance of animal reservoirs for emerging infectious agents. In Liping County of Qiandongnan Autonomous Prefecture, Guizhou Province—a region inhabited by multiple ethnic minorities—the customary consumption of wild rodents persists in certain rural communities. Notably, *Berylmys bowersi* constitutes a predominant species in these dietary practices. This intimate human-rodent interaction substantially elevates the risk of zoonotic transmission, potentially enabling pathogen spillover through both food preparation processes and direct ecological contact.

The high phylogenetic similarity (99.5%) between the isolated strains and the reference *E. marmotae* strain NR_136472.1 supports the notion that these isolates belong to the same species, as confirmed by 16S rRNA gene sequencing and PFGE analysis. Despite the genetic homogeneity of the isolates, the overall genetic diversity of *E. marmotae* within different hosts warrants further exploration, particularly considering the species’ potential for misidentification as *Escherichia coli* (7).

The WGS of strain S2-2 revealed a genome size of 5,037,959 bp with a high-quality assembly, which was basically consistent with the WGS size and GC content of the other three strains we found. The identification of 132 genes associated with antibiotic resistance underscores the potential for *E. marmotae* to acquire and propagate resistance determinants, particularly against commonly used antibiotics like fluoroquinolones, penicillins, cephalosporins, and tetracyclines. This is a significant concern, as multidrug-resistant strains of *E. coli* and *Shigella* spp. are already prevalent in human populations (47–49). Some researchers have isolated *E. marmotae* from crows or wild boars and speculate that they are antibiotic resistant (50–52). The observed resistance profiles in *E. marmotae* suggest that this species may share similar mechanisms of resistance, including efflux pumps and alterations in antibiotic targets, mechanisms commonly seen in other members of the Enterobacteriaceae family (53).

The virulence factor profile of strain S2-2 reveals a broad array of factors linked to pathogenicity, including genes involved in adherence (fimbriae), invasion (flagella), iron uptake (enterobactin), and toxin production (lipooligosaccharide). These factors are consistent with those seen in other pathogenic *Escherichia* spp., which suggests that *E. marmotae* may be capable of causing disease in both humans and animals. The presence of multiple virulence-associated genes, coupled with the ability to survive in the gastrointestinal tract, may contribute to the species’ potential for causing enteric infections or systemic diseases (51, 54). Furthermore, *E. marmotae* is not only classified as a zoonotic pathogen, but it has also been identified in various environmental reservoirs, such as soil and water. This highlights the potential for human infection through environmental exposure to this microorganism (55, 56).

Interestingly, PFGE and WGS comparison using ANI and dDDH analyses confirmed the high genomic similarity between *E. marmotae* and other *Escherichia* spp., especially *E. marmotae* strains. However, the distinction from *E. coli* and *Shigella* spp., supported by low dDDH and ANI values, confirms that *E. marmotae* is a separate and distinct species within the genus *Escherichia*. This distinction is critical when considering diagnostic challenges, as the phenotypic similarities between *E. marmotae* and *E. coli* could lead to misidentification in clinical laboratories (7, 8).

The biochemical characterization of the isolates, which showed distinct fermentation patterns and enzyme activities, further supports the differentiation of *E. marmotae* from other *Escherichia* spp. Notably, the inability to ferment sucrose and produce indole, combined with positive hydrogen sulfide production, provides a unique biochemical signature for *E. marmotae*. These characteristics may be useful for developing diagnostic tools to differentiate *E. marmotae* from other closely related species in clinical settings. Accurate differentiation of *E. marmotae* from closely related *Escherichia* spp. is clinically imperative, as misidentification may lead to erroneous antimicrobial susceptibility interpretations, delayed targeted therapy, and compromised epidemiological surveillance (57, 58). Physiological and biochemical tests, including fermentation of sorbitol and production of acid and gas from glucose, were consistent with the characteristics of *E. coli* reported by Li et al. with negative results in the hydrogen sulfide test (59). However, our results differed from those reported by Liu et al. in tests such as ONPG, IND, VP, and melibiose, where our strains tested positive, while Liu’s strains tested negative (1).

Overall, the results of this study contribute valuable insights into the molecular and biochemical characteristics of *E. marmotae* isolated from *Berylmys bowersi*. The genetic homogeneity, presence of virulence factors, and resistance profiles observed here warrant further research into the ecological role of *E. marmotae* in wildlife, its zoonotic potential, and its capacity to evolve resistance to multiple antibiotic classes. Continued surveillance and molecular characterization of this pathogen are critical for understanding its epidemiology and potential risks to public health.

While this study provides novel insights into the genomic and phenotypic characteristics of *E. marmotae* isolated from *Berylmys bowersi*, several limitations should be acknowledged:

Sample representativeness: the analysis was restricted to four isolates from a single geographic region (Guizhou Province), which may not fully capture the genetic diversity or ecological adaptability of *E. marmotae* across broader host ranges or environmental reservoirs.
Functional validation gaps: although WGS revealed virulence factors and resistance genes, the absence of *in vivo* infection models or clinical outcome data limits our understanding of the pathogen’s actual zoonotic transmission capacity and disease mechanisms.
Plasmid-mediated resistance mechanisms and mobile genetic elements: the role of extrachromosomal elements in resistance gene dissemination remains to be investigated through conjugation assays and long-read plasmid phasing, which should be prioritized in future epidemiological tracking of this emerging pathogen.

### Conclusion

This study is the first to report the isolation of *E. marmotae* from *Berylmys bowersi* and provides an in-depth characterization of its genomic, biochemical, and antibiotic resistance profiles. The findings indicate that *E. marmotae* shares significant genetic similarity with other members of the *Escherichia* genus, particularly *E. marmotae* strains, but can be differentiated based on biochemical and phenotypic characteristics. The presence of multiple virulence factors and antibiotic resistance mechanisms in the isolated strains suggests that *E. marmotae* may pose a potential health risk, especially in wildlife and possibly in humans. Further research is needed to fully assess the pathogenicity and zoonotic potential of this emerging species.

## Data Availability

The full genetic sequence of the four strains has been uploaded to GenBank. The accession number of the 16S rRNA is PV473833(https://www.ncbi.nlm.nih.gov/nuccore/PV473833), and the accession number of the whole-genome sequencing is CP188181-CP188183( https://www.ncbi.nlm.nih.gov/nuccore/CP188181), https://www.ncbi.nlm.nih.gov/nuccore/CP188182), https://www.ncbi.nlm.nih.gov/nuccore/CP188183).

## SUPPLEMENTAL MATERIAL

Supplemental material spectrum.02946-24-s0001.docx Supplemental table captions.

Table S1 spectrum.02946-24-s0002.xlsx The identity value of virulence analysis of the strain S2-2 in the VFDB database.

Table S2 spectrum.02946-24-s0003.xlsx The identity value of resistance gene analysis of strain S2-2 in CARD database.

Table S3 spectrum.02946-24-s0004.xlsx Biochemical test results.

ASM does not own the copyrights to Supplemental Material that may be linked to, or accessed through, an article. The authors have granted ASM a non-exclusive, world-wide license to publish the Supplemental Material files. Please contact the corresponding author directly for reuse.
