# Supplementary material for: Whole-genome characterization and antibiotic resistance phenotype of Escherichia marmotae first isolated from Berylmys bowersi
Source: Microbiol Spectr. 2025 May 28;13(7):e02946-24. doi: 10.1128/spectrum.02946-24 (PMC12211086; doi:10.1128/spectrum.02946-24)
Supplement: Supplemental material — Supplemental table captions. [file spectrum.02946-24-s0001.docx]

**Supplementary table 1.** The identity value of virulence analysis of the strain S2-2 in the VFDB database.

**Supplementary table 2.** The identity value of resistance gene analysis of strain S2-2 in CARD database.

**Supplementary table 3.** Biochemical test results
